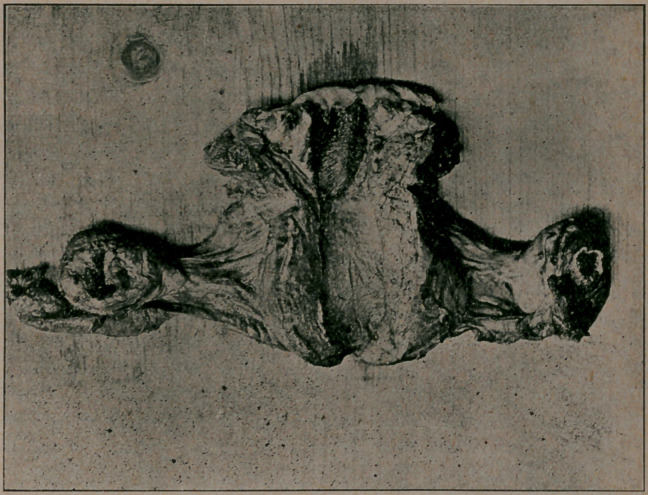# Abscess of Both Ovaries—Rupture and Death

**Published:** 1898-05

**Authors:** B. F. Kingsley

**Affiliations:** San Antonio, Texas


### Abscess of Both Ovaries—Rupture and Death.

BY B. F. KINGSLEY, M. D.

About two years ago, Mrs. A. came to San Antonio three weeks after having produced an abortion on herself with some kind of an instrument. She had pain and tenderness over lower abdomen, with high fever. She was sent to the hospital and curetted, washed and packed twice within the next ten or twelve days, with the effect of improving, for the time, her symptoms.

About two weeks later her symptoms grew worse, and she died rather suddenly. This is the history, about as I was able to obtain it, not having seen her myself.

There being no responsible person at hand, I seized the opportunity to hold an autopsy, with the result of finding the rare specimen presented—a ruptured abscess of both ovaries. It will be seen that the uterus and ovaries are not overly enlarged. The abscesses or ovaries are neither of them large. There were absolutely no adhesions, but a small quantity of sero-purulent fluid in the peritoneal cavity, marked tympanites and peritonitis.

San Antonio, Texas, April 11, 1898.

## Figures and Tables

**Figure f1:**